# Minimum flow decomposition in graphs with cycles using integer linear programming

**DOI:** 10.1007/s10898-025-01556-8

**Published:** 2025-11-24

**Authors:** Fernando H. C. Dias, Lucia Williams, Brendan Mumey, Alexandru I. Tomescu

**Affiliations:** 1 Department of Mathematics and Systems Analysis, Aalto University, 02150 Espoo, Finland; 2 Department of Computer Science, University of Helsinki, 00560 Espoo, Finland; 3 School of Computing, Montana State University, Bozeman, MT USA; 4 Department of Computer Science, University of Montana, Missoula, MT USA

**Keywords:** Network Flow, Flow Decomposition, Integer Linear Programming, Bioinformatics, Transportation Science

## Abstract

Minimum flow decomposition (MFD) — the problem of finding a minimum set of weighted source-to-sink paths that perfectly decomposes a flow — is a classical problem in Computer Science, and variants of it are powerful models in a different fields such as Bioinformatics and Transportation. Even on acyclic graphs, the problem is NP-hard, and most practical solutions have been via heuristics or approximations. While there is an extensive body of research on acyclic graphs, currently there is no *exact* solution on graphs with cycles. In this paper we present the first ILP formulation for three natural variants of the MFD problem in graphs with cycles, asking for a decomposition consisting only of weighted source-to-sink paths or cycles, trails, and walks, respectively. On three datasets of increasing levels of complexity from both Bioinformatics and Transportation, our approaches solve any instance in under 12 minutes. Our implementations are freely available at https://github.com/algbio/MFD-ILP.

## Introduction

### Background

Flow decomposition (FD) is a classical and well-researched network problem in which a source-to-sink flow needs to be decomposed into a set of weighted source-to-sink paths (and possibly cycles) such that their respective weights perfectly fit each edge’s flow. An essential textbook property (see, e.g. [1]) is that any flow in a graph with *m* edges can be decomposed into at most *m* paths or cycles (each with some associated weight). However, some flow paths or cycles could be suitably combined to form a smaller decomposition. Therefore, a popular and practically motivated variant of the flow decomposition problem is to find the decomposition into the *minimum* amount of paths or cycles (Minimum Flow Decomposition problem, MFD). This variant, however, is NP-hard, even if the input network is restricted to a directed acyclic graph (DAG), as first observed by [2].

Despite their NP-hardness, MFD and its variants are used in many applications, such as computer networking [3–6], Transportation science [7–11], and bioinformatics [12–16]. The input graph may naturally be a DAG in some applications, such as reference-based RNA transcript assembly in bioinformatics. However, in many applications, the input network graph naturally contains cycles, as in computer and transportation networks, or sequence assembly problems from bioinformatics [17–19].

Despite the need to decompose flows with cycles in applications, much of the recent progress on MFD has focused on DAGs due to properties associated with acyclicity that can be exploited into algorithms, as in the fixed-parameter tractable (FPT) algorithms of [14] and [20], the integer linear programming (ILP) formulation of [12], and the heuristic algorithm called Catfish [21].

In fact, no exact (that is, returning a truly *minimum* solution) solver exists for MFD on graphs with cycles. Because different applications may require different types of decompositions, in this paper, we study three natural versions of the problem on cyclic inputs: decomposing into source-to-sink paths or cycles (in this paper, paths and cycles do not repeat nodes, except for cycles where the first and last nodes are the same), into source-to-sink trails (which may repeat nodes but not edges), and into source-to-sink walks (which may repeat both nodes and edges).

### Related work

The MFD problem was first formally studied in [2], where it was shown to be NP-hard, even on DAGs. This result was later strengthened by [4], who proved several additional results for the problem. They showed that the problem is also NP-hard when the flow values come from only the set $\{1, 2, 4\}$, that MFD is hard to approximate (i.e., there is some $\epsilon > 0$ such that MFD cannot be approximated within a $(1+\epsilon )$ factor unless P=NP), even on DAGs. On the positive side, on DAGs, it is possible to decompose all but a $\varepsilon$-fraction of the flow within a $O(1/\varepsilon )$ factor of the optimal number of paths. [3] gave an approximation algorithm for DAGs with an exponential approximation factor based on decomposing the flow into paths with weights that are powers of two. Recently, [22] showed that if the weights of the flow decomposition paths can also take negative integer values, then a polynomial-time approximation algorithm exists for DAGs, with an approximation factor of $\lceil \log ||f||\rceil + 1$, where ||*f*|| is the largest flow value of any edge. Additionally, [14] showed that the problem on DAGs is FPT in the size of the minimum decomposition.

In practice, though, applications tend to use heuristics, such as the greedy methods based on choosing the widest or longest paths [2], and these heuristics can be applied to both DAGs and general graphs, possibly with cycles. Some of these greedy methods could be improved on DAGs by making iterative modifications to the flow graph before finding a greedy decomposition, as shown by [21]. However, [22] also showed that there exist some instances (even DAGs) where the MFD has size $O(\log m)$ (*m* is the number of edges of the graph), where such greedy methods return flow decompositions as large as $\Omega (m/\log m)$, meaning that they can be exponentially worse than the optimum.

Many application-oriented algorithms on DAGs for MFD and related problems use ILP, taking advantage of existing software such as Gurobi [23] and CPLEX [24]. However, most of these solutions encode every possible source-to-sink path as a variable, yielding exponential-size ILP formulations that are impractical to solve for larger instances, which occur in applications such as computer networking [5]. A common strategy amongst bioinformatics applications to deal with this issue is to pre-select some subset of the possible paths in the graph, either for all instances (as in vg-flow [25] and CLIIQ [26]), or only when the input is extensive (as in MultiTrans [27] and SSP [28]). However, by pre-selecting only some paths that can be part of a solution, these algorithms may return a non-optimal decomposition.

Recently, two polynomial-size ILP formulations have been proposed that can be used to solve MFD on DAGs without pre-selecting paths. Namely, the program JUMPER [16] requires an additional condition on the input DAG, namely that it has a unique Hamiltonian path. This allows all paths to be uniquely determined by subsets of edges that do not pairwise overlap along the Hamiltonian path, yielding a formulation for decomposing into *k* paths using only a quadratic number of variables and constraints. In [12], the authors give a general solution working on any DAG to find a size *k* decomposition, even without a Hamiltonian path, again using only a quadratic number of variables and a linear number of constraints. The insight is that source-to-sink paths can be encoded using conservation of flow constraints, as in [29]. However, neither of these approaches can be straightforwardly extended to handle non-DAG inputs.

As outlined above, most contributions to the flow decomposition problem have been restricted to DAGs. The only attempts for the general cyclic case that we know of are limited to heuristics. Since these heuristics can be exponentially worse than the optimum (as discussed above), the quest for efficient and exact MFD approaches to the cyclic case is wide open.

### Our contributions

In this paper, we give the first *exact* solutions for MFD on graphs with cycles, based on ILP, for all three natural variants mentioned in Section 1.1. In addition, we show that our solutions are also efficient through experiments on three datasets from two application domains, Bioinformatics and Transportation Science.

While our solutions follow the same high-level approach from [12] when solving MFD on DAGs (i.e., first model each of the *k* paths by an auxiliary unit flow and then require that these paths admit weights so that they form a flow decomposition), the cyclic case poses several difficulties, which we overcome as follows.

As a major novelty, in this paper, we show how to formulate different types of walks in graphs with cycles (i.e., paths *or* cycles, trails, walks) using ILP. The simple path-modelling techniques from [12] heavily rely on the acyclicity of the graph; thus, here, we need to develop new techniques. Another difficulty we overcome is that trails can visit nodes multiple times, and walks can visit both nodes and edges multiple times. Apart from complicating the modelling, these facts also render some constraints more challenging to linearize than in [12]. Our solutions can be summarized as follows:

- For the paths *or* cycles variant, we extend the so-called “sequential formulation” of [30] (see also [29]) to uniformly model either a path *or* a cycle (i.e., by a *single* set of variables with associated constraints). We still model them by an auxiliary unit flow but add new constraints to require that this auxiliary flow induces either a path *or* a cycle.
- For the trails and walks variants, we give a novel reachability-based formulation characterizing when the auxiliary unit flow corresponds to a trail or a walk, as follows. In order to model a source-to-sink walk, more than simply modelling a unit flow is required because in graphs with cycles, it may induce *isolated* strongly connected components. Thus, we add constraints to require that any node incident to the unit flow modelling the walk is reachable from the source via a spanning tree rooted at the source and using edges of the unit flow.
- For the trails variant, we also propose an alternative approach that forbids such isolated strongly connected components via an iterative constraint generation procedure. This approach is inspired by previous constraint generation approaches from [29, 31]. However, these previous approaches model paths by forbidding only cycles (since a unit flow induces a path if and only if it contains no cycles). Trails and walks can contain cycles, so we must forbid all strongly connected components.

All the formulations are of quadratic size in the number of constraints and variables, except for the constraint generation approach, which introduces a number of constraints linear in the graph’s number of edges, per iteration.

Our ILPs constitute the *first* exact solutions for the NP-hard MFD problem on graphs with cycles for its three natural variants on such inputs. At the same time, we also show that they are efficient with biological and transportation data from different sources, solving simple instances in under 60 seconds and more complex ones in under 12 minutes. Lastly, mathematical programming can provide significant flexibility that is difficult to incorporate in purely algorithmic formulations. In fact, our ILP formulations can be easily extended to practical aspects of applications. This can be done either by suitably adapting the formulation of what constitutes an element of a decomposition (here, we extended paths to paths or cycles, trails, and walks) or by adapting the condition of when the weighted paths fit the flow edges (for example, by considering inexact, or imperfect flow decomposition as in [32] and [12]).

The paper is organized as follows. We review the basic concepts of flow networks and flow decomposition and define the problems addressed in Section 2. We next present the ILP models for each problem variant, starting with paths or cycles in Section 3.1, trails in Section 3.2, and walks in Section 3.3. Numerical experiments are provided in Section 4, and concluding remarks are discussed in Section 5.

## Preliminaries

### Basic notions

In this paper, by a graph $G = (V, E)$, we mean a directed graph with $E \subseteq V \times V$. We also assume that all graphs are weakly connected meaning that there is an undirected path between any pair of nodes. A *source* of *G* is a node without incoming edges, and a *sink* is a node without outgoing edges. We will use *n* and *m* to denote the cardinality of the sets of nodes *V* and edges *E*, respectively, of the graph.

A *walk* in *G* is a sequence of edges in *E* where consecutive edges share the end and the start. Given a walk *W* in *G*, and an edge (*u*, *v*) of *G*, we denote by *W*(*u*, *v*) the number of times *W* passes through (*u*, *v*) (and thus 0 if *W* does not pass through (*u*, *v*)). We say that *W* is a *trail* if $W(u,v) = 1$ for all edges (*u*, *v*) in *W*. We say that *W* is a *path* if *W* repeats no node and that *W* is a *cycle* if *W* repeats no node, except that the first and the last node are the same. If *W* has first node *s* and last node *t*, we say that *W* is an *s*-*t* *walk* (or *s*-*t* *trail*, or *s*-*t* *path*, accordingly). A *strongly connected component of* *G* is an inclusion-maximal set *C* of nodes of *G* such that for any $x,y \in C$, there is an *x*-*y* path and an *y*-*x* path.

### Network flows and flow decompositions

#### Definition 1

*(Flow network)* A tuple $G=(V,E,f)$ is said to be a *flow network* if (*V*, *E*) is a graph with unique source *s* and unique sink *t*, where for every edge $(u,v) \in E$ we have an associated positive integer *flow value* $f_{uv}$, satisfying *conservation of flow* for every $v \in V \setminus \{s,t\}$, namely:

1 $$\begin{aligned} \sum _{(u,v) \in E} f_{uv} = \sum _{(v,w) \in E} f_{vw}. \end{aligned}$$

Next, we define when a set of generic walks in a flow network forms a flow decomposition. By considering different types of walks, we will obtain the specific problem variants of this paper.

#### Definition 2

*(k*-*Flow Decomposition)* A *k*-*flow decomposition* $({\mathcal {W}},w)$ for a flow network $G=(V,E,f)$ is a set of *k* walks ${\mathcal {W}}= (W_1,\ldots ,W_k)$ in *G* and associated weights $w = (w_1,\ldots ,w_k)$, with each $w_i \in {\mathbb {Z}}^{+}$, such that for each edge $(u,v) \in E$ it holds that:

2 $$\begin{aligned} \sum _{i \in \{1,\dots ,k\}} w_iW_i(u,v) = f_{uv}. \end{aligned}$$

The number *k* of walks is also called the *size* of the flow decomposition.

**Fig. 1:**
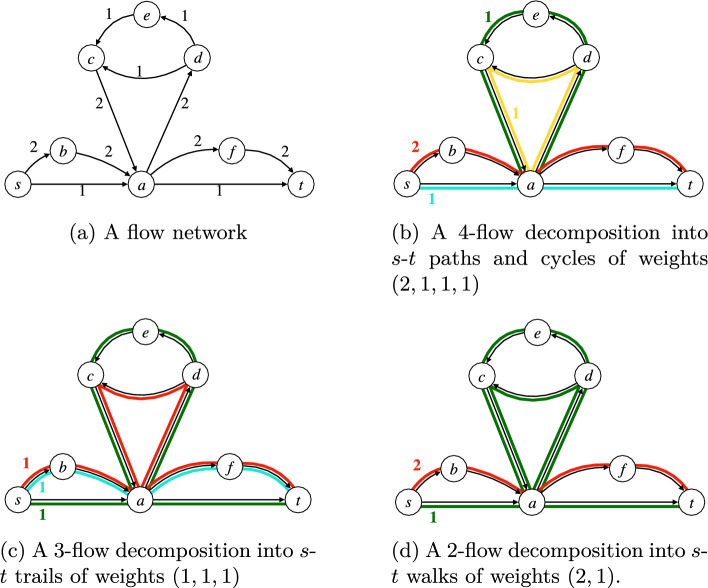
Example of a flow network (1a) and three different types of minimum flow decomposition into paths or cycles (1b), trails (1c) and walks (1d)

We will consider several types of walks and obtain corresponding variants of the flow decomposition problem (see also Figure 1). We will also refer to the walks of decomposition as its *elements*. Next, we define the flow decomposition problems by asking for decompositions into *at most* *k* elements.

#### Definition 3

*(k*-*Flow Decomposition Problems)* Given a flow network $G=(V, E,f)$, consider the problem of finding a flow decomposition into at most *k* walks and associated weights. If these walks are required to be:

- *s*-*t* paths or cycles, then we call the resulting problem *k*-*Flow Decomposition into Paths or Cycles* (*k*-*FDPC)*;
- *s*-*t* trails, then we call the resulting problem *k*-*Flow Decomposition into Trails* (*k*-*FDT)*;
- *s*-*t* walks, then we call the resulting problem *k*-*Flow Decomposition into Walks* (*k*-*FDW)*.

It is a standard result (see e.g. [1]) that any flow network can be decomposed into paths or cycles because one can consider any edge (*u*, *v*), and by conservation of flow, extend this edge in both directions either into an *s*-*t* path, or into a cycle, associate weight 1 to it, and remove it from the flow network, obtaining another flow network. We can analogously deduce that any flow network can also be decomposed into a collection of *s*-*t* walks. The most straightforward proof of this fact can be obtained by creating another graph by replacing every edge (*u*, *v*) with $f_{uv}$ parallel edges and adding as many parallel edges from *t* to *s* as there is flow outgoing from *s*. Since in this graph, every node has as many in-neighbours as out-neighbours (by construction and by conservation of flow), the resulting graph has an Eulerian walk with the first and last node equal to *s*. Every time this walk passes through one of the parallel edges from *t* to *s*, we cut it by removing such edges. The resulting pieces are *s*-*t* walks covering all edges, thus forming a flow decomposition of the original graph, where each walk is associated with weight 1.

**Fig. 2:**
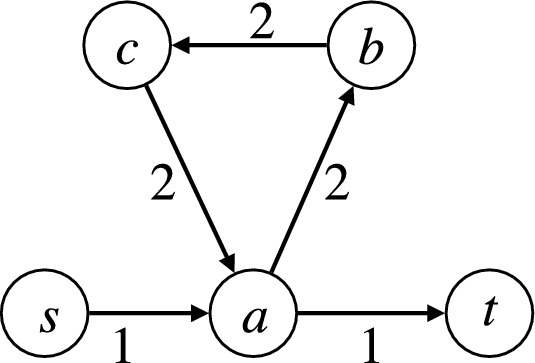
Example of a network that does not admit a decomposition into *s*-*t* trails

However, not all flow networks admit flow decomposition into *s*-*t* trails. For example, this is the case for the flow network made up of edges (*s*, *a*), (*a*, *b*), (*b*, *c*), (*c*, *a*), (*a*, *t*), with flow values $f_{sa} = 1$, $f_{ab} = 2$, $f_{bc} = 2$, $f_{ca} = 2$ and $f_{ut} = 1$ (see also Figure 2).

In practice, we are interested in decompositions of minimum size (i.e., with the minimum number of elements, that is, walks, recall Definition 2). As such, we can introduce the *minimization* version of each problem as follows.

#### Definition 4

*(Minimum Flow Decomposition (MFD))* For each of the problems *k*-FDPC, *k*-FDT, *k*-FDW, its *minimization* version asks for finding a flow decomposition of minimum size (i.e., minimizing *k*).

All our ILP formulations will assume *k* paths/cycles, trails, or walks at most. As such, the minimization versions of the problems can be solved via an iterative process, trying all possible *k* in increasing order until reaching the minimum size $k^*$ of decomposition (or *m*, which is an upper bound on the size of an MFD in all problem variants, as discussed above). If no decomposition into trails is found for $k \le m$, then there exists no such decomposition. This iterative approach leads to $k^*$ runs of our ILPs.

However, a more efficient approach, leading to only $O(\log k^*)$ runs of our ILPs, is based on the fact that if a decomposition of size at most *k* exists, then trivially also one of size at most $k' > k$ exists as well. As such, we can first do a *doubling* (or *exponential*) approach [33] by running our ILPs with $k = 1, 2, 4, \dots$, until finding the smallest *k* such that a decomposition of size at most 2*k* exists, but one of size *k* does not exist. Then, it holds that $k^*\in (k,2k]$, and we can find $k^*$ with a linear search in this interval.

### ILP formulation of *s*-*t* paths in DAGs and of flow decompositions

In this section, we review the formulation from [29] of an *s*-*t* path in a graph $G = (V, E)$, with $s,t \in V$, and then recall the use of this formulation in the ILP solution for MFD in DAGs [12].

For every edge $(u,v) \in E$, we can introduce a binary variable $x_{uv}$. The idea is to represent the *s*-*t* path by the edges (*u*, *v*) having $x_{uv} = 1$. If *G* is a DAG, it suffices to impose the following constraint, stating that the *s*-*t* path starts with a single edge outgoing from *s*, ends with a single edge in-coming to *t*, and at every other node *v*, either it does not pass through the node, or if it reaches *v*, it also exits *v*. Since *G* is a DAG, no walk can use a node *v* multiple times.

3 $$\begin{aligned} \sum _{(u,v) \in E} x_{uv} - \sum _{(v,u) \in E} x_{vu} = {\left\{ \begin{array}{ll} 0, &  \text {if }v \in V \setminus \{s,t\}, \\ 1, &  \text {if }v = t, \\ -1, &  \text {if }v = s. \end{array}\right. } \end{aligned}$$

We will refer to the above constraint as the “flow conservation” constraint (not to be mistaken with the conservation of the input flow). In [12], a flow decomposition in DAGs into exactly *k* *s*-*t* paths is expressed by adding *k* copies of the above variables, namely $x_{uvi}$, for $i \in \{1,\dots ,k\}$, and imposing the flow conservation constraint (3) for all of them. The flow decomposition constraint (2) can be stated as

4 $$\begin{aligned}&\sum _{i \in \{1,\dots ,k\}} x_{uvi}w_i = f_{uv},  &   \forall (u,v) \in E. \end{aligned}$$

We refer to this ILP formulation for MFD in DAGs as the *standard formulation* (${min} k$-FD). The complete ILP formulation, which we give as Model 26 can be found in Appendix A. Our formulations will build on this, mainly by possibly changing Eq. (3) and adding further constraints to the $x_{uvi}$ variables to model all types of walks considered in this paper.

#### Remark 1

The constraint in Eq. (4) can be linearized by introducing an integer variable $\pi _{uvi}$ representing the product $x_{uvi}w_i$, together with the following constraints, where *M* is any constant strictly greater than the right-hand side of the first inequality in Eq. (5b):

5a $$\begin{aligned}  &    &f_{uv} = \sum _{i \in \{1,\dots ,k\}} \pi _{uvi},  &   \forall (u,v) \in E, \end{aligned}$$

5b $$\begin{aligned}  &    &\pi _{uvi} \le M x_{uvi},  &   \forall (u,v) \in E, \forall i \in \{1,\dots ,k\}, \end{aligned}$$

5c $$\begin{aligned}  &    &\pi _{uvi} \le w_i,  &   \forall (u,v) \in E, \forall i \in \{1,\dots ,k\}, \end{aligned}$$

5d $$\begin{aligned}  &    &\pi _{uvi} \ge w_i - (1-x_{uvi})M,  &   \forall (u,v) \in E, \forall i \in \{1,\dots ,k\}. \end{aligned}$$

Indeed, in inequality (5b), for $x_{uvi}=1$, the constraint should be bounded by the flow value $f_{uv}$. If $x_{uvi}=0$, then the constraint is not binding. Therefore, the left side can be any value smaller or equal to zero. For constraint (5d), when $x_{uvi}=1$, the value of *M* is irrelevant; when $x_{uvi}=0$, the constraint is not binding as long as *M* is a positive number. Therefore, the best value of *M* is $M = f_{uv}$.

## ILP formulations for MFD in graphs with cycles

### *k*-Flow decomposition into paths or cycles

In this section, we tackle the *k*-FDPC problem. We start as in the standard formulation by using the same binary variables $x_{uvi}$ for every edge $(u,v) \in E$ and every $i \in \{1,\dots ,k\}$. Since paths and cycles can visit any node at most once, we adapt the flow conservation constraint (3) into the following constraints:

6a $$\begin{aligned}  &    &\sum _{(u,v) \in E} x_{uvi} \le 1  &   \forall u \in V, \forall i \in \{1, \ldots , k\}, \end{aligned}$$

6b $$\begin{aligned}  &    &\sum _{(u,v) \in E} x_{uvi} - \sum _{(v,w) \in E} x_{vwi} = 0  &   \forall i \in \{1, \ldots , k\}, \forall v \in V \setminus \{s, t\}. \end{aligned}$$

However, if *G* is not a DAG, the binary variables $x_{uvi}$ could, for example, induce an *s*-*t* path and one or more cycles (not even reachable from the *s*-*t* path).

To overcome this, we start from a formulation of an *s*-*t* path in general graphs, presented in [29] as the *sequential formulation*, and attributed to [30]. This formulation introduces a nonnegative integer variable $d_v$ for every node $v \in V$, which will stand for the position of *v* in the path. The following constraint is introduced for every edge:

7 $$\begin{aligned} d_v \ge d_u + 1 + (n-1)(x_{uv} - 1)  &   \forall (u,v) \in E. \end{aligned}$$

This assigns a partial order to the nodes, such that for any edge (*u*, *v*) with $x_{uv} = 1$, we have $d_u < d_v$. We can also consider the $d_v$ variables as a *pseudo-distance* function, where the distance from *s* to *t* is obtained by minimizing $d_t$. However, constraint (7) as is forbids cycles altogether, which is not the goal in the *k*-FDPC problem.

**Fig. 3:**
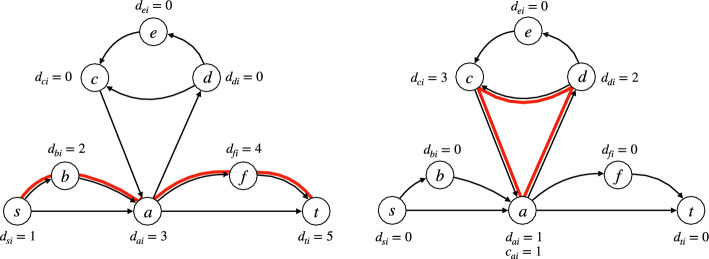
Example of the edge variables $x_{uvi}$ (where we draw edge (*u*, *v*) in red if $x_{uvi} = 1$, and in black otherwise), satisfying Eqs. (6b) and (6a). On the left, the assignments of the variables $d_{vi}$ shown in the figure (and $c_{vi} = 0$ for all $v \in V$) satisfy constraint (8). On the right, the assignments of the variables $d_{vi}$ shown in the figure (and $c_{vi} = 0$ for all $v \in V \setminus \{a\}$, and $c_{ai} = 1$) satisfy constraint (8). Note that the only edge (*u*, *v*) such that $d_{vi} < d_{ui}$ is (*c*, *a*). However, constraint (8) holds for (*c*, *a*) because $c_{ai} = 1$

In order to handle both paths and cycles, we modify the sequential formulation constraint (7) as follows. For every $v \in V \setminus \{s,t\}$, we introduce a binary variable $c_{vi}$ to denote whether node *v* is a “start of cycle” node in the *i*-th element of the flow decomposition. We also introduce positive integer variables $d_{vi}$, and change (7) into the following constraint:

8 $$\begin{aligned} d_{vi} \ge d_{ui} + 1 + (n-1)(x_{uvi} - 1 - c_{vi})  &   \forall (u,v) \in E, \forall i \in \{1,\ldots ,k\}. \end{aligned}$$

Namely, this constraint imposes that either a node is the starting point of a cycle or its order in the path is always increasing. Finally, we impose:

9 $$\begin{aligned} \sum _{v\in V} x_{svi} + \sum _{v\in V} c_{vi} \le 1  &   \forall i \in \{1,\dots ,k\}. \end{aligned}$$

That is, for every element *i* of the decomposition, either there is some edge (*s*, *v*) with $x_{svi} = 1$ (in which case we have just an *s*-*t* path, and no cycle), or $c_{vi} = 1$ for some *v* (or none of these hold) (see Figure 3). Because of constraint (6b), if $\sum _{v\in V} x_{svi} = 1$, then the binary $x_{uvi}$ variables induce just an *s*-*t* path and no other cycles. If $c_{vi} = 1$, this means that we have no *s*-*t* path and just one cycle (or none at all, if all $x_{uvi}$ variables are zero). Finally, observe that adding constraint (9) we obtain that constraint (6a) is always satisfied, since otherwise, we have either at least two *s*-*t* paths passing through *u* in some element *i* of the decomposition (and thus $\sum _{v\in V} x_{svi} \ge 2$), or two cycles passing through *u* in some element *i* of the decomposition (and thus $\sum _{v\in V} c_{vi} \ge 2$).

The complete ILP formulation for this problem variant (referred from now on as *min* k-FDPC), which we give as Model 27 in B, has $O((|V|+|E|)k)$ variables and constraints.

#### Remark 2

The formulation for *k*-FDPC presented here can decompose the flow into up to *k* paths or cycles. However, one can quickly obtain a formulation where there are *exactly* *k* elements in the decomposition, as follows. The constraint in (9) allows the number of total cycles and paths up to *k* by allowing either term of the left-hand side to be at most 1. Therefore, this constraint needs to be changed to:

10 $$\begin{aligned} \sum _{v\in V} x_{svi} + \sum _{v\in V} c_{vi} = 1  &   \forall i \in \{1,\dots ,k\}. \end{aligned}$$

### *k*-Flow decomposition into trails via constraint generation

In this section, we tackle the next problem, *k*-FDT, namely finding a *k*-flow decomposition into at most *k* *s*-*t* trails. Recall from the previous sections that the binary *x* variables induce a flow. The only case when such flow does not correspond to an *s*-*t* trail is when it has a strongly connected component that is not reachable from *s* or, equivalently, does not reach *t*.

We start with a simple lemma stating that, due to flow conservation, this global reachability property can be reduced to checking whether any strongly connected component *C* induced by the edges of the trail has an edge outgoing from *C* (i.e., an edge (*u*, *v*) with $x_{uvi} = 1$, such that *u* appears in *C*, but (*u*, *v*) does not belong to *C*). For a strongly connected component *C* of *G*, we denote by *E*(*C*) the set of edges of *C* and by $\delta ^+(C)$ the set of edges (*u*, *v*) such that *u* belongs to *C*.

#### Lemma 1

Let $G = (V, E)$ be an arbitrary graph with source and sink nodes *s* and *t*, respectively. Let *W* be a set of edges of *G*, where for every edge $(u,v) \in E$ we define $W(u,v) := 1$, if $(u,v) \in W$, and $W(u,v) := 0$ otherwise. It holds that the edges of *W* can be ordered to obtain a *s*-*t* trail passing through each edge of *W* if and only if the following conditions hold:

1. For every $$v \in V$$, $$\displaystyle \sum _{(u,v) \in E} W(u,v) - \sum _{(v,u) \in E} W(v,u) = {\left\{ \begin{array}{ll} 0, &  \text {if }v \in V \setminus \{s,t\}, \\ 1, &  \text {if }v = t, \\ -1, &  \text {if }v = s. \end{array}\right. }$$
2. For any strongly connected component *C* of the subgraph of *G* induced by the edges in *W* and different from $\{t\}$, the set $\left( \delta ^+(C) \setminus E(C)\right) \cap W$ is non-empty.

#### Proof

For the forward implication, suppose the edges of *W* can be ordered to obtain an *s*-*t* trail *T* that satisfies condition 1. To see that *W* satisfies condition 2., let *C* be a strongly connected component of the subgraph of *G* induced by the edges of *W*, different from $\{t\}$. First, note that node *t*, being a sink, does not belong to *C*.

If *C* contains a single node, say $v \ne t$, then the edge of the trail outgoing from *v* belongs to $\left( \delta ^+(C) \setminus E(C)\right) \cap W$. Otherwise, let (*u*, *v*) be the edge of *C* that is last in the order given by the trail *T*. Say that (*v*, *w*) is the edge following (*u*, *v*) in *T* (which exists because $v \ne t$). We have that $(v,w) \in \delta ^+(C)$ (since *v* belong to *C*), $(v,w) \notin E(C)$ (since (*u*, *v*) is the last edge of *C* in the order given by *T*, and $(u,v) \in W$, since (*u*, *v*) is an edge of the trail. Thus, the set $\left( \delta ^+(C) \setminus E(C)\right) \cap W$ is non-empty, and *W* satisfies Condition 2.

**Fig. 4:**
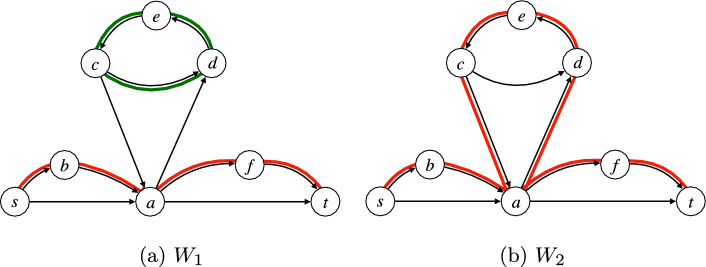
The graph from Figure 1, with the edge (*d*, *c*) reversed. This creates the cycle (*c*, *d*, *e*, *c*) in the graph, which can lead to a set $W_1$ of edges (on the left, with edges in both red and green), satisfying condition 1. of Lemma 1, but with a strongly connected component (in green) violating condition 2. of Lemma 1. Instead, the set $W_2$ (on the right, with edges in red) satisfies both conditions of Lemma 1

For the reverse implication, suppose *W* satisfies the two conditions. Since the values *W*(*u*, *v*) induce a flow in *G*, it remains to prove that this flow is decomposable into a *single* *s*-*t* trail. Since $\sum _{(u,t) \in E} W(u,t) - \sum _{(t,u) \in E} W(s,u) = 1$, the only way this does not hold is when *W* also contains a set of edges forming a strongly connected component, and no node in this component reaches *t*. This means that all edges outgoing from the nodes of *C* are to other nodes of *C* (by flow conservation and by the fact that *t* is not reachable from *C*). However, this contradicts the second condition. See the example in Figure 4. $\square$

As in the standard formulation, for every edge $(u,v) \in E$ and for every $i \in \{1,\dots ,k\}$, we introduce a binary variable $x_{uvi}$ that will equal 1 if and only if the *s*-*t* trail $W_i$ passes through (*u*, *v*) (since trails can also visit edges at most once). The first condition of Lemma 1 is the same as the flow conservation condition in Equation (3). However, as in the previous formulation, since we are modelling up to *k* trails, we impose it in its weaker form below. (At the end of this section (Remark 5), we explain how to model *exactly* *k* trails.)

11 $$\begin{aligned}  &    &\sum _{(s,v) \in E} x_{svi} \le 1  &   \forall i \in \{1, \ldots , k\}, \end{aligned}$$

12 $$\begin{aligned}  &    &\sum _{(u,v) \in E} x_{uvi} - \sum _{(v,w) \in E} x_{vwi} = 0  &   \forall i \in \{1, \ldots , k\}, \forall v \in V \setminus \{s, t\}. \end{aligned}$$

To model the second condition of Lemma 2, a first inefficient method would be to first exhaustively enumerate possible strongly connected subgraphs of *G*; their number is finite since *G* has a finite number of nodes. For each such strongly connected subgraph *C*, add constraints to prevent it from not reaching *t* via the second condition of Lemma 1. The following constraint models the second condition of Lemma 2 for a given strongly connected component *C*, where |*C*| denotes the number of edges of *C* (see Remark 3 on how to linearize this constraint):

13 $$\begin{aligned}&\sum _{(u,v) \in E(C)} x_{uvi} = |C| \rightarrow \sum _{(u,v) \in \delta ^+(C) \setminus E(C)} x_{uvi} \ge 1  &   \forall i \in \{1,\dots ,k\}. \end{aligned}$$

In order to avoid exhaustively enumerating all possible strongly connected subgraphs *C* of *G* and imposing constraint (13) for each such *C*, we can consider the following iterative procedure, in which constraint (13) is not imposed at the beginning, but it is iteratively imposed for any strongly connected component induced by the variables $x_{uvi}$ not satisfying the constraint.

**Fig. 5:**
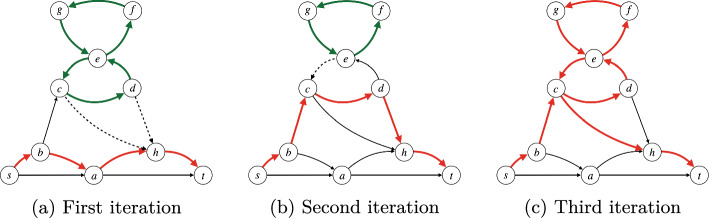
Illustration of the iterative process described in Algorithm 1. The edges in the red form an *s*-*t* trail while the green ones form a strongly connected component that does not reach *t* via edges of the trail (i.e. not satisfying constraint (13)). We assume that the flow conservation and flow decomposition constraints are valid in all three iterations. In iteration one (Figure 5a), there is a strongly connected component (in green). Based on the constraints imposed in Eq. (13), at least one of the outgoing edges from the strongly connected component (dashed edges) must be selected. In Figure 5b, the original strongly connected component no longer exists after imposing those constraints. However, a new one is present in the solution, and the same set of constraints must also be imposed for it. In Figure 5c , the solution corresponds to an *s*-*t* trail, where no isolated strongly connected component is present

More precisely, we can consider a parameterized version of our ILP denoted as *k*-FDC(${\mathcal {C}}$) made up of the constraints in Eq. (4),(11),(12),(13), where ${\mathcal {C}}$ is a set of strongly connected components for which constraint (13) is imposed. Initially, ${\mathcal {C}}$ is empty. For any strongly connected component *C* induced by the variables $x_{uvi}$ not satisfying (13), we add *C* to ${\mathcal {C}}$ and rerun the ILP. This procedure eventually stops because the number of strongly connected subgraphs of *G* is finite, as mentioned above. We summarize this procedure also as Algorithm 1 (see also Figure 5). This iterative addition of constraints to an initial formulation is known in the literature as a *constraint generation* (or *cut generation*), and it is based on the cycle elimination technique first proposed by [31] for the Traveling Salesman Problem.

The full ILP formulation for *k*-FDT(${\mathcal {C}}$), which we give as Model 28 in C, has $O((|E|+|{\mathcal {C}}|)k)$ variables and $O((|V|+|V|+|{\mathcal {C}}|)k)$ constraints.

#### Remark 3

In order to linearize (13), we first rewrite it as the following disjunction:

14 $$\begin{aligned}&\sum _{(u,v) \in E(C)} x_{uvi} \le |C| - 1 \vee \sum _{(u,v) \in \delta ^+(C) \setminus E(C)} x_{uvi} \ge 1  &   \forall C \in {\mathcal {C}}, \forall i \in \{1,\dots ,k\}. \end{aligned}$$

To linearize this disjunction, we introduce the binary variables $\beta _{Ci}$, for the strongly connected component *C*, and each $i \in \{1,\dots ,k\}$. Following standard techniques (as applied in Remark 1), let *M* be any constant strictly greater than the right-hand side of the first inequality in (14). We can rewrite (14) as the following three constraints:

15a $$\begin{aligned}&\sum _{(u,v) \in E(C)} x_{uvi} \ge |C| - M(1-\beta _{Ci})  &   \forall C \in {\mathcal {C}}, \forall i \in \{1,\dots ,k\}, \end{aligned}$$

15b $$\begin{aligned}&\sum _{(u,v) \in E(C)} x_{uvi} - |C| + 1 - M\beta _{Ci} \le 0  &   \forall C \in {\mathcal {C}}, \forall i \in \{1,\dots ,k\}, \end{aligned}$$

15c $$\begin{aligned}&\sum _{(u,v) \in \delta ^+(C) \setminus E(C)} x_{uvi} \ge \beta _{Ci}  &   \forall C \in {\mathcal {C}}, \forall i \in \{1,\dots ,k\}, \end{aligned}$$

15d $$\begin{aligned}&\beta _{Ci} \in \{0,1\}  &   \forall C \in {\mathcal {C}}, \forall i \in \{1,\dots ,k\}. \end{aligned}$$

For *M*, a suitable value would be $M = |C|$. In Eq. (15a) and Eq. (15b), the left side of each constraint should not be bounded when $\beta _{Ci}=1$ and $\beta _{Ci}=0$, respectively. Hence, the smallest number that guarantees this condition is $M = |C|$.

#### Remark 4

The check-in Algorithm 1 for strongly connected components not satisfying constraint (13) can be implemented efficiently (in linear time), as follows. Consider the graph $G'$ where we add all the edges (*u*, *v*) of *G* where the $x_{uvi}$ variables are set to 1, plus the additional edge (*t*, *s*). Compute the strongly connected components of $G'$, doable in linear time [34]. Using arguments as in the proof of Lemma 1, one can easily see that $G'$ has only one strongly connected component if and only if the check-in Algorithm 1 is false (i.e., the $x_{uvi}$ variables do not induce any strongly connected component *C* violating (13)).

**Algorithm 1:**
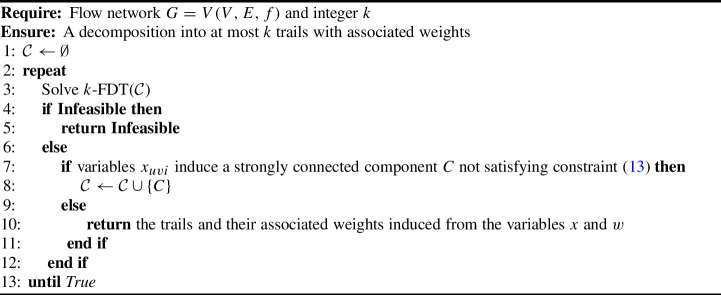
Algorithm for *k*-Flow Decomposition into Trails via Constraint Generation (${min} k$-FDT (Model))

#### Remark 5

In order to allow the formulation presented in this section to obtain a *k*-flow decomposition into *exactly* *k* *s*-*t* trails, we need to change the flow conservation constraints in Equation (12) and Equation (11) into its basic form as in Equation (3):

16 $$\begin{aligned} \sum _{(u,v) \in E} x_{uvi} - \sum _{(v,u) \in E} x_{vui} = {\left\{ \begin{array}{ll} 0, &  \text {if }v \in V \setminus \{s,t\}, \forall i \in \{1,\dots ,k\} \\ 1, &  \text {if }v = t, \forall i \in \{1,\dots ,k\}\\ -1, &  \text {if }v = s, \forall i \in \{1,\dots ,k\}. \end{array}\right. } \end{aligned}$$

### *k*-Flow decomposition into trails and walks

In this section, we give a formulation that works for both problems *k*-FDT and *k*-FDW. Our formulations will be based on the following characterization of an *s*-*t* walk: all its nodes must be reachable from *s*, using only the edges of the *s*-*t* walk.

#### Lemma 2

Let $G = (V, E)$ be an arbitrary graph with source and sink nodes *s* and *t*, respectively. Let *W* be a multiset of edges of *G*, where for every edge $(u,v) \in E$, we denote by *W*(*u*, *v*) the number of times (*u*, *v*) appears in the multiset *W*. It holds that the edges of *W* can be ordered to obtain a *s*-*t* walk passing *W*(*u*, *v*) times through each edge (*u*, *v*) if and only if the following conditions hold:

1. For every $$v \in V$$, $$\displaystyle \sum _{(u,v) \in E} W(u,v) - \sum _{(v,u) \in E} W(v,u) = {\left\{ \begin{array}{ll} 0, &  \text {if }v \in V \setminus \{s,t\}, \\ 1, &  \text {if }v = t, \\ -1, &  \text {if }v = s. \end{array}\right. }$$
2. For every node $v \in V$ appearing in some edge of *W*, there is an *s*-*v* path using only edges in *W* (i.e. *v* is reachable from *s* using edges in *W*).

#### Proof

The forward implication is immediate: any *s*-*t* walk satisfies the first condition of the lemma, and every node *v* appearing in some edge of *W* is reachable from *s* via an *s*-*v* path obtained by removing all cycles of the walk between *s* and *v*.

**Fig. 6:**
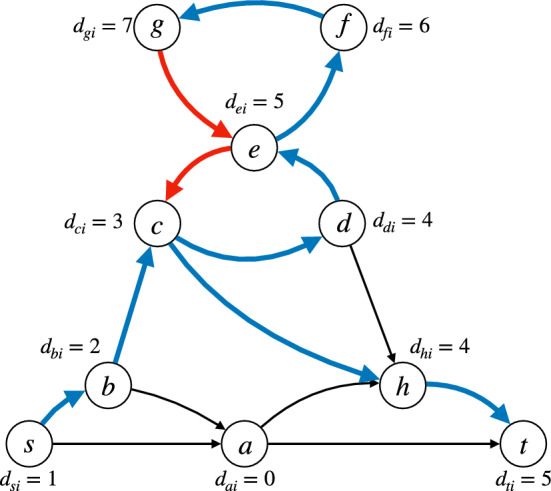
Illustration of Lemma 3. Thick edges (*u*, *v*) are those for which $x_{uvi} = 1$. An edge (*u*, *v*) is drawn in blue if $y_{uvi} = 1$ (these form a spanning tree of the selected nodes rooted at *s*) and in red otherwise. Next to each node *v*, we draw the $d_{vi}$ value. Note that any blue edge is also thick (condition (19a)), any selected node *v* has exactly one incoming blue edge (condition (19b)), and for any selected node *v* its in-coming blue edge (*u*, *v*) satisfies $d_{ui} < d_{vi}$ (condition (19c))

For the reverse implication, we argue as in the proof of Lemma 1. Suppose *W* satisfies the two conditions. Since the values *W*(*u*, *v*) induce a flow in *G*, it remains to prove that this flow is decomposable into a *single* *s*-*t* walk. Since $\sum _{(u,s) \in E} W(u,s) - \sum _{(s,u) \in E} W(s,u) = -1$, the only way this does not hold is when *W* also contains a set of edges forming a strongly connected component, and no node in this component is reachable from *s*. However, this contradicts the second condition. See Figure 6. $\square$

For every edge $(u,v) \in E$ and for every $i \in \{1,\dots ,k\}$, we introduce a nonnegative integer variable $x_{uvi}$ now representing the number of times the *s*-*t* walk $W_i$ passes through (*u*, *v*). Since walks can use edges arbitrarily often, we impose $x_{uvi} \in {\mathbb {Z}}^+ \cup 0$. Imposing $x_{uvi} \in \{0,1\}$ will suffice to obtain the formulation for trails because a trail can visit every edge at most once.

As in the case of trails from the previous section, we impose the flow conservation condition of Lemma 2 in its weaker form (as in Equation (11) and Equation 12):

17a $$\begin{aligned}  &    &\sum _{(s,v) \in E} x_{svi} \le 1  &   \forall i \in \{1, \ldots , k\}, \end{aligned}$$

17b $$\begin{aligned}  &    &\sum _{(u,v) \in E} x_{uvi} - \sum _{(v,w) \in E} x_{vwi} = 0  &   \forall i \in \{1, \ldots , k\}, \forall v \in V \setminus \{s, t\}. \end{aligned}$$

The second condition of Lemma 2 can be encoded as follows. We say that an edge $(u,v) \in E$ is *selected* by $W_i$ if $x_{uvi} \ge 1$, and that a node *v* is *selected* by $W_i$ if *v* has a selected edge in-coming to it (i.e. $x_{uvi} \ge 1$, for some $(u,v) \in E$).

For every $v \in V$ and every $i \in \{1,\dots ,k\}$, we introduce a non-negative integer variable $d_{vi}$. These variables generalize the corresponding variables from the *k*-FDPC problem. The idea is to impose that $d_{vi}=0$ if and only if node *v* is *not selected* in walk $W_i$. Thanks to the conservation-of-flow condition (17), we can model this as:

18a $$\begin{aligned}&d_{si} = 1  &   \forall i \in \{1,\dots ,k\}, \end{aligned}$$

18b $$\begin{aligned}&\sum _{(u,v) \in E} x_{uvi} = 0 \rightarrow d_{vi} = 0  &   \forall v \in V \setminus \{s\}, \forall i \in \{1,\dots ,k\}. \end{aligned}$$

Moreover, for every selected node *v*, we want to guarantee that *v* is reachable from *s* using selected edges. This holds if and only if there is some selected edge (*u*, *v*) such that *u* is reachable from *s*. We will thus impose that every selected node *v* has a least one in-coming selected edge (*u*, *v*) such that $d_{vi} \ge d_{ui} + 1$ (these will correspond to a spanning tree of the selected nodes rooted at the source). We encode selected incoming edges, giving the strict increase with a binary variable $y_{uvi}$ for every edge. The variable $y_{uvi}$ equals 1 for exactly one selected in-coming edge to each selected node $v \in V \setminus \{s\}$. We thus state the following constraints (see Figure 6 for an example), for all $i \in \{1,\dots ,k\}$:

19a $$\begin{aligned}&y_{uvi} = 1 \rightarrow x_{uvi} \ge 1  &   \forall (u,v) \in E, \end{aligned}$$

19b $$\begin{aligned}&\left( \sum _{(u,v) \in E} x_{uvi} \ge 1\right) \rightarrow \left( \sum _{(u,v) \in E} y_{uvi} = 1\right)  &   \forall v \in V \setminus \{s\} \end{aligned}$$

19c $$\begin{aligned}&\left( \sum _{(u,v) \in E} x_{uvi} \ge 1\right) \rightarrow \left( \sum _{(u,v) \in E} y_{uvi}(d_{vi} - d_{ui}) \ge 1\right)  &   \forall v \in V \setminus \{s\}. \end{aligned}$$

The following lemma states that Eqs. (17) to (19) correctly model *s*-*t* walks.

#### Lemma 3

The ILP made up of equations (17), (18) and (19), is feasible if and only if conditions 1 and 2 from Lemma 2 hold.

#### Proof

For the forward direction, for every selected *v*, the condition in Eq. (19c) guarantees that we have a selected edge (*u*, *v*), such that $d_{vi} \ge d_{ui} + 1$. Thus, following all such edges until *s*, we obtain an *s*-*v* path.

For the reverse direction, for every $v \in V$, set $d_{vi}$ to be the number of nodes of a shortest *s*-*v* path using only edges (*u*, *v*) for which $x_{uvi} \ge 1$, or 0 if there is no such path. $\square$

Next, we explain how to transform the conditional structures in constraints (18) and (19). The second condition in (18) can be expressed as:

20 $$\begin{aligned}&\sum _{(u,v) \in E} x_{uvi} \ge d_{vi}  &   \forall v \in V \setminus \{s\}, \forall i \in \{1,\dots ,k\}. \end{aligned}$$

Similarly, the first and third conditions in (19) can be expressed as

21 $$\begin{aligned}&x_{uvi} \ge y_{uvi}  &   \forall (u,v) \in E, \forall i \in \{1,\dots ,k\}, \end{aligned}$$

22 $$\begin{aligned}&\sum _{(u,v) \in E} x_{uvi} \le \sum _{(u,v) \in E} y_{uvi}(d_{vi} - d_{ui})  &   \forall v \in V \setminus \{s\}, \forall i \in \{1,\dots ,k\}. \end{aligned}$$

For the second condition in (19), we can apply a similar, but slightly modified, approach using the fact that $\sum _{(u,v) \in E} x_{uvi} \le |E|$:

23a $$\begin{aligned}&\sum _{(u,v) \in E} x_{uvi} \le |E| \sum _{(u,v) \in E} y_{uvi}  &   \forall v \in V \setminus \{s\}, \forall i \in \{1,\dots ,k\}, \end{aligned}$$

23b $$\begin{aligned}&\sum _{(u,v) \in E} y_{uvi} \le 1  &   \forall v \in V \setminus \{s\}, \forall i \in \{1,\dots ,k\}. \end{aligned}$$

#### Remark 6

In order to linearize Equation 22, note first that $y_{uvi}$ is a binary variable. Next, we can introduce a new integer variable $d_{uvi} = d_{vi} - d_{ui}$. Note that *n* is an upper bound for any $d_{vi}$ (since the interval [0, *n*] for each $d_{vi}$ variable is sufficiently large to allow for a feasible solution if there is any), and thus we have that each $d_{uvi} \in [-n,n]$. As such, the product $y_{uvi}d_{uvi}$ can be linearized as in Remark 3 (except that now we have a negative lower bound).

24a $$\begin{aligned}&\phi _{uvi} \le M y_{uvi}  &   \forall (u,v) \in E, \forall i \in \{1,\dots ,k\}, \end{aligned}$$

24b $$\begin{aligned}&\phi _{uvi} \ge -M y_{uvi}  &   \forall (u,v) \in E, \forall i \in \{1,\dots ,k\}, \end{aligned}$$

24c $$\begin{aligned}&\phi _{uvi} \le (d_{vi} - d_{ui}) + (1-y_{uvi})M  &   \forall (u,v) \in E, \forall i \in \{1,\dots ,k\}, \end{aligned}$$

24d $$\begin{aligned}&\phi _{uvi} \ge (d_{vi} - d_{ui}) - (1-y_{uvi})M  &   \forall (u,v) \in E, \forall i \in \{1,\dots ,k\}. \end{aligned}$$

#### Remark 7

For walks, the flow superposition constraint in Equation 4 involves the product of two integer variables (since, for walks, $x_{uvi}$ is not assumed to be binary). Thus, we cannot simply linearize this product using Remark 1 since that requires one of the variables to be binary. However, we can reduce this more general case to several applications of Remark 1, using a *power-of-two technique*. In this technique, one of the integer variables is replaced by its expression as a sum of powers of two by introducing an auxiliary binary variable for each power of two (smaller than the maximum value it can achieve). More specifically, suppose we want to linearize the product *xy* between two integer variables *x* and *y*. Suppose also that $x \in [{\underline{x}},{\overline{x}}]$, with $0 \le {\underline{x}}$. For each $j \in \{0,\dots ,\lfloor \log _2({\overline{x}})\rfloor \}$, we introduce a binary variable $x_j$ and add the constraint

$$\begin{aligned}x = \sum _{j \in \{0,\dots ,\lfloor \log _2({\overline{x}})\rfloor \}}2^jx_j.\end{aligned}$$

The product *xy* then becomes

$$\begin{aligned}\sum _{j \in \{0,\dots ,\lfloor \log _2({\overline{x}})\rfloor \}}2^jx_jy.\end{aligned}$$

Each product $x_jy$ is now between the binary variable $x_j$ and the integer variable *y*, which can now be linearized as in Remark 1.

The complete ILP formulation for this problem variant, which we give as Model 29 in D (referred as ${min} k$-FDW when $x_{uvi}$ is treated as binary variables) and as Model 30 in E (referred as ${min} k$-FDT (Model) when $x_{uvi}$ is treated as integer variables).

#### Remark 8

In order to obtain a *k*-flow decomposition into *exactly* *k* *s*-*t* walk, we can make the same change as in Remark 5, by imposing the basic flow conservation constraint:

25 $$\begin{aligned} \sum _{(u,v) \in E} x_{uvi} - \sum _{(v,u) \in E} x_{vui} = {\left\{ \begin{array}{ll} 0, &  \text {if }v \in V \setminus \{s,t\}, \forall i \in \{1,\dots ,k\}\\ 1, &  \text {if }v = t, \forall i \in \{1,\dots ,k\}\\ -1, &  \text {if }v = s, \forall i \in \{1,\dots ,k\}. \end{array}\right. } \end{aligned}$$

The value of the constant *M* should be large enough to guarantee that the left side of the constraints in (6) are satisfied adequately. Hence, in constraint (24a), when $y_{uvi}=1$, the maximum value for $\phi _{uvi}$ should be equal to the maximum value for $d_{uvi}$ (i.e. ${\overline{d}}_{uvi}$). In constraint (24d), when $y_{uvi}=1$, $\phi _{uvi}$ should be at most lower bounded by zero, and that can be achieved by using $M = {\overline{d}}_{uvi}$.

For walks, where $y_{uvi}$ and $x_{uvi}$ behave as integer variables, this product can be linearized as in Remark 7.

## Experiments

### Experiment design

#### Solvers

We designed our experiments to test the minimization versions of the three problem variants from Definition 3. For ${min} k$-FDPC, we implemented the model from Section (3.1) (in full as Model 27 in B). For ${min} k$-FDT, we implemented both the iterative constraint generation approach described in Section 3.2 (in full as Model 28 in C) and the model from Section 3.3 (in full as Model 29 in D) with binary $x_{uvi}$ variables. For ${min} k$-FDW, we implemented the model from Section 3.3 (in full as Model 29 in D) with $x_{uvi}$ as non-negative integer variables. To find the minimum *k*, we implemented two different search algorithms for a minimum *k*: linear increment and doubling increment as described at the end of Section 2.2. All four were implemented using the Python API for CPLEX 20.1 under default settings. We ran our experiments on a personal computer with 16 GB of RAM and an Apple M1 processor at 2.9 GHz.

#### Datasets

We test the performance of the solvers under a range of biological and transportation graph topologies and flow weights, which we also make available at https://github.com/algbio/MFD-ILP. As our first dataset, we took one of the larger datasets produced by [35] (rnaseq/sparse_quant_SRR020730.graph) and used in several flow decomposition benchmarking studies [14, 20]. This contains transcriptomic data as a flow in a DAG for each human genome gene. A slight alteration was applied to this dataset to create cycles in each instance. We call this dataset **SRR020730-Salmon-Adapted**.

We collected instances from flow network transportation data curated by [36] for the second dataset. We refer to this as **Transportation Data**. The dataset comprises network flows from different cities worldwide collected through various scenarios.

To obtain a more complex dataset, we created a dataset consisting of genome graphs from up to 50 variants of *E. coli* genomes, with flow values coming from the abundances of these genomes. The complete dataset is composed of more than a thousand instances with a different number of genomes. For the sake of this experiment, we sampled up to 10 graphs from each number of genomes, starting from 2. The graphs in this dataset are the largest.

These three datasets cover a range of real-world applications indicative of our models’ efficiency and scalability in such scenarios. Since the precise details of these datasets are outside this paper’s scope, we provide a more detailed description of how the datasets were created at https://github.com/algbio/MFD-ILP.

### Results and discussion

**Fig. 7:**
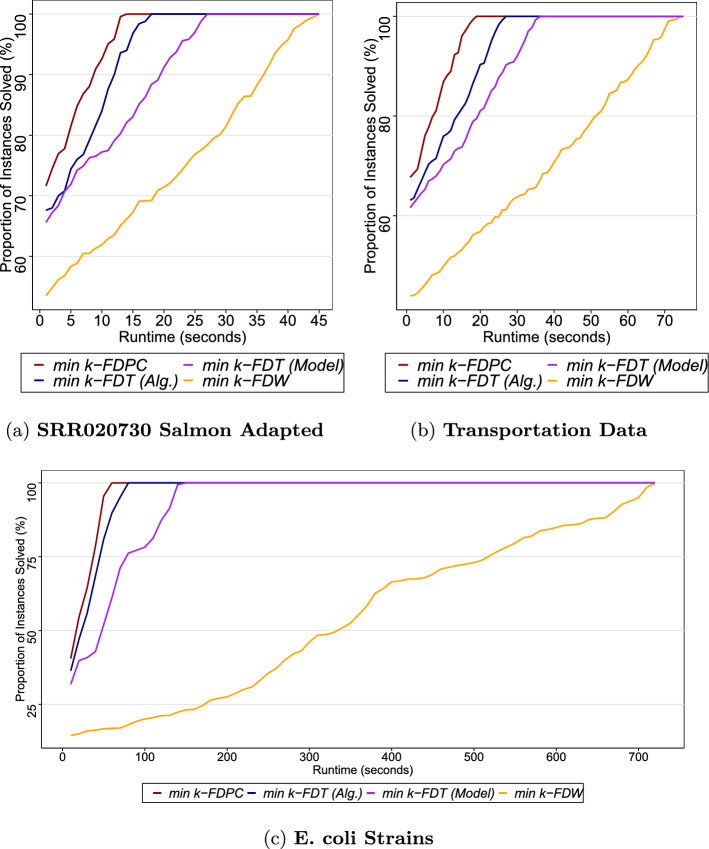
The percentage of all instances (y-axis) individually solvable within a certain number of seconds (x-axis)

The numerical results are displayed in Tables 1 and 2 as well in Figure 7. Figure 7 summarizes the performance of the models in terms of runtime by showing the proportion of all instances that can be (individually) solved within a certain time. Overall, we observe that all four formulations are efficient, finishing in under 45 seconds on the first dataset, 75 seconds on the second dataset, and 1.5 minutes on the third dataset. Note that the datasets are increasingly complex in terms of the number of nodes and edges. Moreover, the runtime of ${min} k$-FDPC is relatively smaller than that for ${min} k$-FDT and ${min} k$-FDW. This is expected, given that the number of constraints and variables is smaller when handling paths and cycles.

We also evaluate how the models scale in terms of the complexity of the instances of each dataset. We focus on the size *k* of the flow decomposition since the models have a linear size of *k*. Table 1 shows a comparative experiment on the performance of three formulations (${min} k$-FDPC, *min* *k*-FDT (both algorithm and model version) and *min* *k*-FDW) presented in the paper. For the three tools, the results are from instances grouped by the number of *k* paths as output by *min* *k*-FDPC. We also report the average number of nodes and edges (|*V*| and |*E*|) and the average runtime per graph (under the name of each method and in seconds). Note that the reported runtime is the total required to solve each instance after completing the iterative process. For *min* *k*-FDT, we also report the number of iterations required by Algorithm 1 and the runtime reflects the runtime required throughout all iterations (including due to the addition of more constraints in the constraint generation process). Table 2 compared the number of paths, cycles, trails and walks reported by each formulation individually. In Table 1, we group the graphs into ranges based on the minimum size of a flow decomposition into paths or cycles since this is the most basic problem variant (column *min* *k*). Since |*V*| and |*E*| also increase as *min* *k* increases, this table also indicates how the models scale regarding graph sizes. The runtimes of all models do scale up adequately, reiterating that they are viable candidates for real applications. The reachability model for trails (where its main decision variables $x_{uvi}$ are binary) scales better than for walks (where these variables are integer).

**Table 1:** Averages and standard deviations (in parentheses) for graph size, running times of the four variants of the problem, and the number of iterations of Algorithm 1 until a feasible solution is found. For Algorithm 1, the runtime is the total over all iterations. If an instance did not admit a decomposition into trails, we do not include it in the runtime average for $$min $$ *k*-FDT. The increment in in *k* is done linearly until a feasible solution is found

					$$min $$ *k*-FDPC (sec)	$$min $$ *k*-FDT (sec)			$$min $$ *k*-FDW (sec)
	*min* *k*	$\#$instances	|*V*|	|*E*|		Algorithm 1	$\#$iter	Model 29	
SRR020730 Salmon Adapted	4-10	5131	23.42 (4.21)	45.12 (8.22)	3.22 (0.36)	5.13 (0.52)	2.32	10.2 (1.41)	13.42 (1.12)
	11-15	595	32.34 (3.42)	60.21 (5.41)	4.43 (0.93)	9.13 (1.23)	2.94	14.5 (1.54)	19.88 (3.41)
	16-20	236	43.81 (4.67)	71.14 (9.57)	8.68 (1.72)	14.03 (2.12)	3.20	17.2 (2.72)	28.61 (5.74)
	21-*max*	60	51.42 (5.23)	102.14 (11.31)	12.42 (2.41)	18.12 (3.34)	4.51	27.2 (4.41)	39.79 (5.81)
Transportation Data	4-10	200	43.22 (6.21)	78.21 (10.13)	4.51 (0.56)	8.32 (1.13)	2.41	16.54 (2.12)	28.22 (3.31)
	11-15	150	50.74 (7.51)	83.21 (7.65)	7.13 (1.32)	15.21 (2.41)	3.52	21.33 (3.23)	53.15 (6.13)
	16-20	50	64.33 (4.78)	93.21 (5.11)	10.42 (2.13)	17.89 (3.29)	5.93	28.92 (4.09)	78.61 (7.21)
	21-*max*	20	80.32 (5.21)	130.1 (3.13)	14.54 (1.21)	24.72 (2.12)	6.32	36.94 (3.21)	112.71 (6.88)
E. coli Strains	4-10	80	48.21 (5.41)	82.13 (7.24)	8.51 (2.41)	14.13 (3.13)	4.33	22.41 (5.31)	56.12 (18.25)
	11-15	50	63.73 (11.42)	90.22 (12.31)	9.24 (3.13)	17.31 (4.41)	5.92	35.38 (10.24)	147.21 (27.13)
	16-20	50	78.34 (22.14)	108.01 (15.44)	17.23 (4.62)	25.89 (7.13)	6.24	53.33 (14.41)	297.35 (42.34)
	21-25	50	91.41 (28.13)	140.13 (17.62)	24.46 (8.42)	30.43 (9.31)	7.51	61.42 (22.14)	412.32 (58.42)
	26-30	50	100.81 (33.32)	156.32 (24.54)	36.93 (10.13)	45.13 (13.41)	8.43	78.43 (29.43)	523.52 (58.21)
	31-*max*	75	120.42 (30.34)	176.42 (30.32)	60.61 (12.42)	79.41 (18.31)	10.21	144.31 (34.62)	657.64 (63.55)

Notice that the scalability in terms of *min* *k* was an issue for MFD algorithms on DAGs before the ILP of [12] because the previous exact FPT algorithm of [14] had an exponential dependency on *min* *k*. Our results imply that also for cyclic graphs, ILP-based solutions have no issues in scaling to larger values of *min* *k*. Thus, even if an FPT algorithm for MFD in graphs with cycles (parameterized by the minimum size of a solution) is open, our results suggest that such an FPT algorithm might not be necessary for practice.

To compare the three problem variants, we study each decomposition type’s minimum size in Table 2. Since a decomposition with trails does not always exist, we also show the percentage of instances admitting such a decomposition. While all instances for all three datasets always admit a decomposition into trails for $min~k \le 15$, for larger *min* *k* values, up to 10%, 5%, and 24% of the instances in the first, second and third datasets, respectively, do not admit such a decomposition. On average, we observe that the minimum solution size for ${min} k$-FDPC is more significant than for ${min} k$-FDT, which is more prominent for ${min} k$-FDW. This is explained by the fact that a trail may use a node multiple times, and a walk may also use an edge numerous times. As such, a trail or a walk may combine a source-to-sink path with one or more cycles if their weights can be appropriately adjusted to form a flow decomposition. All these insights show a clear difference between the problem (as also discussed in Section 1), and all the different variants may be explored in applications.

**Table 2:** Average minimum solution sizes for the flow decomposition problem into path or cycles (also split into the average number of paths and cycles), into trails (and the percentage of instances admitting a flow decomposition into trails), and into walks

		$$min $$ *k*-FDPC			$$min $$ *k*-FDT		$$min $$ *k*-FDW
	*min* *k*	$\#$paths	$\#$cycles	$\#$path or cycles	$\#$trails	$\%$feasible	$\#$walks
SRR020730 Salmon Adapted	4-10	5.1	4.4	9.5	3.5	100	2.8
	11-15	7.1	6.2	13.3	5.1	100	4.3
	16-20	9.7	8.1	17.7	5.8	93	5.4
	21-*max*	13.1	10.3	23.3	10.1	90	9.8
Transportation Data	4-10	5.4	3.2	8.6	4.4	100	3.4
	11-15	6.1	4.4	10.5	5.9	100	4.8
	16-20	8.2	7.3	15.5	7.3	98	5.4
	21-*max*	10.1	8.3	18.4	8.8	95	4.8
E. coli Strains	4-10	5.3	4.9	10.2	4.5	100	3.1
	11-15	8.9	4.4	13.2	8.2	100	6.8
	16-20	10.5	6.3	16.8	9.1	100	7.8
	21-25	15.2	8.5	23.7	12.6	90	9.9
	26-30	19.3	7.1	26.4	15.1	84	12.4
	31-*max*	23.1	11.4	34.5	20.1	76	19.6

Finally, we analyze the two solutions for ${min} k$-FDT, constraint generation (Model 28) and Model 29 with variables $x_{uvi}$ binary. While both are efficient, the former is generally faster than the latter because it has fewer constraints, and on our data, it also requires a small number of iterations (at most 11). However, there is no guarantee that it will be concluded after a small number of iterations in general (but as mentioned in Section 3.2, it does find a feasible decomposition after a finite number of iterations). Another advantage provided by the constraint generation formulation is that algorithmic verification procedures can assist it (in the same way as we delegated the strong connectivity check to an algorithm) and use this to craft more straightforward constraints in the original model (in our case, simple constraints based on out-going edges).

Although no heuristics has adequately been introduced in the literature, [2] has proposed two variations of Dijkstra’s algorithm that can be applied to flow decomposition methods in cyclic graphs. Such implementation were compared to our ${min} k$-FDPC, and the results are displayed in Table 3.

**Table 3:** Comparison of the performance of $$min $$ *k*-FDPC and two heuristic. Both heuristics are derived from ideas present in [2]. For each heuristic, their runtime and difference in terms of a minimal number of paths and cycles are reported. For $$min $$ *k*-FDPC, the increment in *k* is done linearly until a feasible solution is found

			$$min $$ *k*-FDPC (Runtime)	$${Heuristic I}$$ (sec)		$${Heuristic II}$$ (sec)	
	*min* *k*	$\#$instances		Runtime (sec)	Diff.	Runtime (sec)	Diff.
SRR020730 Salmon Adapted	4-10	5131	2.14 (0.16)	0.84 (0.31)	2.81	0.81 (0.41)	2.83
	11-15	595	3.31 (0.45)	1.12 (0.78)	4.21	1.42 (0.93)	3.41
	16-20	236	7.12 (1.22)	2.14 (1.12)	4.15	2.21 (2.42)	4.13
	21-*max*	60	9.43 (1.98)	3.89 (2.41)	5.67	3.12 (2.13)	6.24
Transportation Data	4-10	200	3.09 (0.25)	0.83 (0.13)	2.33	1.04 (0.41)	2.13
	11-15	150	6.31 (1.42)	0.89 (0.23)	2.41	1.93 (0.63)	2.34
	16-20	50	9.25 (2.03)	1.42 (0.68)	4.42	2.41 (1.15)	3.17
	21-*max*	20	11.48 (1.16)	3.21 (1.31)	5.13	5.13 (2.78)	5.12
E. coli Strains	4-10	80	7.48 (1.11)	1.43 (0.41)	3.14	1.22 (0.63)	3.52
	11-15	50	8.66 (2.31)	3.43 (0.91)	4.13	3.25 (0.76)	4.31
	16-20	50	15.12 (3.22)	4.22 (1.31)	5.51	5.61 (1.41)	6.02
	21-25	50	21.45 (7.41)	5.12 (1.41)	6.41	6.61 (2.41)	7.21
	26-30	50	31.31 (8.31)	5.67 (2.41)	7.31	6.89 (4.38)	8.45
	31-*max*	75	48.12 (10.22)	6.89 (3.41)	8.51	8.91 (4.51)	9.59

In Table 3, we compared the runtime of the two heuristics (${Heuristic I}$ and ${Heuristic II}$) to our ${min} k$-FDPC. In ${Heuristic I}$, a shortest path variant is used to determine the paths, while in ${Heuristic II}$, a maximum flow variant is used. Our ILP formulations provide exact solutions (i.e., non-heuristic), and as such, optimal solutions. We used the number of paths and cycles found using this solver as a reference to categorize the instances. For ${Heuristic I}$ and ${Heuristic II}$, we implemented the proposed algorithm presented in [2], where the authors suggest two different algorithms based on simple alterations of Dijkstra’s algorithm. More details can be found in their paper. The runtime of these heuristics is reported, as well as the difference between the number of paths and cycles proposed as solutions from the heuristics and the exact solver.

Based on those results, we can conclude that both heuristics are very efficient regarding runtime, where they can solve all instances in less than 10 seconds. However, regarding their accuracy, both heuristics have some severe drawbacks. As the number of optimal paths and cycles increases, their accuracy drops even further. Although ${min} k$-FDPC is slower than both heuristics, the improvement that those provide does not overwhelm the exact model, which, in our opinion, remains the most resourceful alternative, providing a reasonable trade-off between accuracy and runtime.

For our first set of results, the dataset regarding *E. coli* strains were created using windows length of 2000. Those windows correspond to a segment of the strains used to create such graphs. In the following table, we explore four additional scenarios (with windows length changing from 4000, 8000, 16000 to 32000) to express how far our models can be pushed and some limitations.

**Table 4:** Averages and standard deviations (in parentheses) for graph size, running times of the four variants of the problem, and the number of iterations of Algorithm 1 until a feasible solution is found. For Algorithm 1, the runtime is the total over all iterations. If an instance did not admit a decomposition into trails, we do not include it in the runtime average for $$min $$ *k*-FDT. The increment in *k* is done linearly until a feasible solution is found. All dataset corresponds to variations of the *E. coli* samples

					$$min $$ *k*-FDPC (sec)	$$min $$ *k*-FDT (sec)			$$min $$ *k*-FDW (sec)
	*min* *k*	$\#$instances	|*V*|	|*E*|		Algorithm 1	$\#$iter	Model 29	
E. coli Strains - 4000	4-10	80	56.21 (10.58)	92.31 (10.43)	10.39 (4.13)	12.13 (4.78)	5.56	32.41 (14.42)	180.09 (38.56)
	11-15	50	73.43 (24.22)	100.22 (31.14)	12.67 (6.41)	29.31 (5.53)	7.69	55.38 (20.93)	289.93 (57.32)
	16-20	50	85.34 (32.94)	128.42 (59.40)	27.42 (7.12)	35.89 (6.94)	8.78	73.33 (34.51)	334.20 (62.79)
	21-25	50	104.53 (24.41)	140.41 (62.92)	44.94 (10.92)	40.43 (10.95)	9.41	81.42 (42.54)	552.42 (48.81)
	26-30	50	149.24 (42.53)	136.31 (45.04)	66.31 (13.42)	65.13 (18.51)	10.51	98.43 (47.43)	735.80 (35.93)
	31-*max*	75	299.23 (43.68)	231.42 (30.48)	90.14 (18.24)	129.41 (28.42)	12.13	234.31 (49.32)	841.21 (83.02)
E. coli Strains - 8000	4-10	80	65.34 (14.72)	102.03 (11.43)	13.02 (5.31)	14.13 (3.41)	6.43	64.61 (16.93)	280.95 (48.91)
	11-15	50	78.43 (25.21)	142.59 (32.14)	22.42 (5.51)	30.31 (5.92)	8.01	96.45 (41.37)	389.32 (87.26)
	16-20	50	90.34 (38.67)	131.09 (53.40)	47.42 (8.04)	41.89 (9.47)	9.91	93.03 (42.53)	434.40 (21.75)
	21-25	50	121.53 (20.13)	203.09 (52.92)	54.09 (12.22)	42.43 (13.05)	12.18	121.42 (24.55)	652.53 (88.93)
	26-30	50	130.24 (41.64)	236.51 (31.04)	76.16 (10.61)	89.13 (21.52)	20.50	148.52 (59.63)	835.42 (95.51)
	31-*max*	75	320.23 (45.43)	431.24 (25.48)	100.44 (13.42)	152.41 (35.22)	22.43	304.14 (31.45)	1041.55 (63.42)
E. coli Strains - 16000	4-10	80	78.21 (12.21)	142.22 (33.38)	30.93 (10.93)	42.96 (6.34)	7.42	109.92 (24.51)	480.09 (38.56)
	11-15	50	89.54 (28.85)	160.01 (42.35)	52.54 (15.53)	59.42 (14.53)	8.29	145.49 (30.31)	589.93 (57.32)
	16-20	50	123.47 (39.41)	208.42 (32.31)	77.93 (27.39)	85.03 (16.44)	10.05	135.21 (44.42)	734.20 (62.79)
	21-25	50	144.02 (32.48)	256.31 (41.70)	94.50 (30.05)	100.41 (20.35)	12.52	202.45 (52.42)	952.42 (48.81)
	26-30	50	189.83 (40.94)	267.51 (34.75)	106.13 (43.24)	125.53 (28.15)	14.12	239.62 (57.72)	1435.80 (35.93)
	31-*max*	75	329.60 (64.35)	452.33 (37.42)	150.49 (58.42)	189.63 (37.24)	16.34	344.15 (67.25)	1773.21 (83.02)
E. coli Strains - 32000	4-10	80	96.32 (20.21)	186.40 (31.42)	60.39 (12.04)	72.42 (7.30)	10.52	102.42 (24.42)	539.92 (68.56)
	11-15	50	103.47 (14.85)	203.35 (42.54)	82.40 (26.13)	99.61 (15.35)	13.29	167.32 (30.93)	894.51 (97.24)
	16-20	50	135.72 (42.31)	241.91 (28.43)	97.29 (35.92)	105.93 (26.56)	16.83	273.52 (54.15)	1354.02 (122.99)
	21-25	50	204.04 (44.14)	403.52 (34.53)	124.42 (44.28)	140.33 (39.02)	19.13	281.94 (82.42)	2589.74 (48.85)
	26-30	50	229.53 (72.32)	367.14 (51.43)	186.92 (52.23)	265.95 (48.95)	20.15	348.94 (87.91)	3093.87 (50.35)
	31-*max*	75	439.23 (62.81)	531.23 (40.54)	294.46 (74.93)	329.64 (68.23)	23.91	334.28 (109.23)	3491.13 (183.23)

In Table 4, the results concerning *E. coli* with different window lengths is displayed. With longer windows, the dimension of the graphs increases accordingly, resulting in longer runtimes for the formulations. This decrease in performance is considerable when the window length goes beyond 8000 and 32000, where the average runtime reaches around 30 minutes and around 1 hour, respectively. Nevertheless, the runtime increases linearly in the window length (but not in the graph size), and ultimately, our formulations are the first optimal solvers for the problems. Although solutions for those instances exist, the runtime highlights the weakness of our formulation, which struggles with scalability to larger instances. However, our models are novel and are the first attempt to solve this problem optimally.

The final analysis we conducted was a change in how the increment in *k* is done. In the previous examples, all the changes were done in a single increment. In the following table, the increment was doubled from its previous value until a feasible solution was found.

**Table 5:** Averages and standard deviations (in parentheses) for graph size, running times of the four variants of the problem, and the number of iterations of Algorithm 1 until a feasible solution is found. For Algorithm 1, the runtime is the total over all iterations. If an instance did not admit a decomposition into trails, we do not include it in the runtime average for $$min $$ *k*-FDT. The increment in *k* is done by doubling its previous value until a feasible solution is found

					$$min $$ *k*-FDPC (sec)	$$min $$ *k*-FDT (sec)			$$min $$ *k*-FDW (sec)
	*min* *k*	$\#$instances	|*V*|	|*E*|		Algorithm 1	$\#$iter	Model 29	
SRR020730 Salmon Adapted	4-10	5131	23.42 (4.21)	45.12 (8.22)	2.14 (0.16)	4.31 (0.22)	2.32	9.24 (0.98)	11.25 (0.82)
	11-15	595	32.34 (3.42)	60.21 (5.41)	3.31 (0.45)	6.49 (0.81)	2.94	10.22 (1.03)	15.71 (1.90)
	16-20	236	43.81 (4.67)	71.14 (9.57)	7.12 (1.22)	11.25 (1.36)	3.20	14.23 (1.68)	23.42 (3.27)
	21-*max*	60	51.42 (5.23)	102.14 (11.31)	9.43 (1.98)	13.21 (2.14)	4.51	18.23 (3.41)	33.91 (4.39)
Transportation Data	4-10	200	43.22 (6.21)	78.21 (10.13)	3.09 (0.25)	7.25 (0.93)	2.41	12.41 (1.89)	24.09 (1.93)
	11-15	150	50.74 (7.51)	83.21 (7.65)	6.31 (1.42)	11.31 (1.61)	3.52	20.08 (2.16)	46.51 (3.39)
	16-20	50	64.33 (4.78)	93.21 (5.11)	9.25 (2.03)	13.42 (2.94)	5.93	25.27 (3.65)	59.78 (4.18)
	21-*max*	20	80.32 (5.21)	130.1 (3.13)	11.48 (1.16)	20.25 (1.01)	6.32	31.43 (1.78)	98.15 (5.86)
E. coli Strains	4-10	80	48.21 (5.41)	82.13 (7.24)	7.48 (1.11)	12.41 (2.80)	3.13	20.12 (4.51)	56.41 (17.02)
	11-15	50	63.73 (11.42)	90.22 (12.31)	8.66 (2.31)	14.31 (3.41)	4.22	31.25 (9.42)	136.11 (24.52)
	16-20	50	78.34 (22.14)	108.01 (15.44)	15.12 (3.22)	23.65 (5.21)	5.12	50.31 (12.42)	281.52 (38.31)
	21-25	50	91.41 (28.13)	140.13 (17.62)	21.45 (7.41)	25.78 (8.52)	6.13	56.78 (18.18)	392.90 (47.51)
	26-30	50	100.81 (33.32)	156.32 (24.54)	31.31 (8.31)	42.42 (11.42)	7.41	72.31 (27.37)	481.42 (51.43)
	31-*max*	75	120.42 (30.34)	176.42 (30.32)	48.12 (10.22)	67.31 (14.14)	9.83	121.42 (31.32)	527.65 (49.51)

In Table 5, the results are very similar to result in Table 1. The only significant change is the corresponding runtime for each model. Although an improvement was observed across different instances and datasets, it does not justify the more complicated implementation for a negligible benefit. Nevertheless, it is important to highlight that such improvement can be significant if combined with the total runtime of all instances across both experiments.

## Conclusions and future work

Flow decomposition is a common problem in various fields of science, including Computer Science, Bioinformatics, and Transportation. Throughout the literature, considerable effort has gone into solving this problem, especially on acyclic inputs. This is because acyclicity guarantees strong properties that can be used to develop algorithms (fixed-parameter tractability, approximation). However, for graphs that contain cycles, an exact solution has yet to be proposed, the few current approaches being heuristics based on greedy algorithms.

This paper considers three natural variants of the flow decomposition problem in graphs with cycles: decompositions into paths or cycles, trails, and walks, respectively. Our ILP formulations generally adopt the same strategy as in the acyclic case from [12], namely formulating what constitutes an element of decomposition and requiring that these weighted elements fit the input flow values. However, the novelty of our formulations resides in modelling the different types of walks in a cyclic graph, which is notably more involved than formulating a path in a DAG. These formulations can also be of independent interest outside the flow decomposition problem since they model basic graph-theoretic notions.

Our formulations have also been extensively tested on biological and transportation datasets. Despite the problems being NP-hard, they solve any instance of the three datasets in under 45 seconds, 75 seconds, and 12 minutes, respectively. Nevertheless, since these are the first exact solutions to the problem on cyclic graphs, the quest for more efficient solutions remains open.

As future work, it would be interesting to extend the models presented here to include other aspects of empirical data, such as all the flow decomposition problem variants discussed in [12] for Bioinformatics applications. More specifically, in one problem variant, we are also given a set of paths (called *subpath constraints*) that must appear in at least one walk of the flow decomposition. The solution from [12] for this variant can be easily adapted to the ${min} k$-FDPC problem (because edges are not repeated), but it remains open how to adapt it to trails or walks because they can repeat edges. In the *inexact* and *imperfect* flow decomposition variants from [12], the weighted walks do not need to fit the input flow perfectly. However, the constraints modelling such solutions from [12] immediately carry over to all our problem variants. Lastly, our reachability formulation can also handle problem variants in which the solution walks can pass through an edge at most a given number of times by just setting a bound to each $x_{uvi}$ variable.

## Full ILP formulation for *k*-Flow decomposition into paths (*k*-FD)

26a $$\begin{aligned}&\mathbf {\forall i \in \{1, \ldots , k\}:} \nonumber \\&\quad \sum _{(s,v) \in E} x_{svi} = 1 \end{aligned}$$

26b $$\begin{aligned}&\quad \sum _{(u,t) \in E} x_{uti} = 1 \end{aligned}$$

26c $$\begin{aligned}&\quad \sum _{(u,v) \in E} x_{uvi} - \sum _{(v,w) \in E} x_{vwi} = 0  &   \forall v \in V \setminus \{s, t\}, \end{aligned}$$

26d $$\begin{aligned}&f_{uv} = \sum _{i \in \{1,\dots ,k\}} \pi _{uvi}  &   \forall (u,v) \in E, \end{aligned}$$

26e $$\begin{aligned}&\mathbf {\forall (u,v) \in E, \forall i \in \{1,\dots ,k\}:} \nonumber \\&\quad \pi _{uvi} \le f_{uv} x_{uvi} \end{aligned}$$

26f $$\begin{aligned}&\quad \pi _{uvi} \le w_i \end{aligned}$$

26g $$\begin{aligned}&\quad \pi _{uvi} \ge w_i - (1-x_{uvi})f_{uv} \end{aligned}$$

26h $$\begin{aligned}&w_i \in {\mathbb {Z}}^+ \end{aligned}$$

26i $$\begin{aligned}&x_{uvi} \in \{0,1\}  &   \forall (u,v) \in E, \forall i \in \{1,\dots ,k\}, \end{aligned}$$

26j $$\begin{aligned}&\pi _{uvi} \in {\mathbb {Z}}^+ \cup \{0\}  &   \forall (u,v) \in E, \forall i \in \{1,\dots ,k\}. \end{aligned}$$

## Full ILP formulation for *k*-Flow decomposition into paths or cycles (*k*-FDPC)

27a $$\begin{aligned}&\mathbf {\forall i \in \{1, \ldots , k\}:} \nonumber \\&\quad \sum _{(u,v) \in E} x_{uvi} \le 1  &   \forall u \in V, \end{aligned}$$

27b $$\begin{aligned}&\quad \sum _{(u,v) \in E} x_{uvi} - \sum _{(v,w) \in E} x_{vwi} = 0  &   \forall v \in V \setminus \{s, t\}, \end{aligned}$$

27c $$\begin{aligned}&f_{uv} = \sum _{i \in \{1,\dots ,k\}} \pi _{uvi}  &   \forall (u,v) \in E, \end{aligned}$$

27d $$\begin{aligned}&\mathbf {\forall (u,v) \in E, \forall i \in \{1,\dots ,k\}:} \nonumber \\&\quad \pi _{uvi} \le f_{uv} x_{uvi} \end{aligned}$$

27e $$\begin{aligned}&\quad \pi _{uvi} \le w_i \end{aligned}$$

27f $$\begin{aligned}&\quad \pi _{uvi} \ge w_i - (1-x_{uvi})f_{uv} \end{aligned}$$

27g $$\begin{aligned}&\mathbf {\forall i \in \{1, \ldots , k\}:} \nonumber \\&\quad d_{vi} \ge d_{ui} + 1 + (n-1)(x_{uvi} - 1 - c_{vi})  &   \forall (u,v) \in E, \end{aligned}$$

27h $$\begin{aligned}&\quad \sum _{v\in V} x_{svi} + \sum _{v\in V} c_{vi} \le 1 \end{aligned}$$

27i $$\begin{aligned}&w_i \in {\mathbb {Z}}^+ \end{aligned}$$

27j $$\begin{aligned}&x_{uvi} \in \{0,1\}  &   \forall (u,v) \in E, \forall i \in \{1,\dots ,k\}, \end{aligned}$$

27k $$\begin{aligned}&\pi _{uvi} \in {\mathbb {Z}}^+ \cup \{0\}  &   \forall (u,v) \in E, \forall i \in \{1,\dots ,k\}, \end{aligned}$$

27l $$\begin{aligned}&c_{vi} \in \{0,1\}  &   \forall v \in V, \forall i \in \{1,\dots ,k\}, \end{aligned}$$

27m $$\begin{aligned}&d_{vi} \in {\mathbb {Z}}^+  &   \forall v \in V, \forall i \in \{1,\dots ,k\}. \end{aligned}$$

## Full ILP formulation for *k*-Flow decomposition into trails via constraint generation (*k*-FDT(${\mathcal {C}}$))

28a $$\begin{aligned}&\mathbf {\forall i \in \{1, \ldots , k\}:} \nonumber \\&\quad \sum _{(s,v) \in E} x_{svi} \le 1 \end{aligned}$$

28b $$\begin{aligned}&\quad \sum _{(u,v) \in E} x_{uvi} - \sum _{(v,w) \in E} x_{vwi} = 0  &   \forall v \in V \setminus \{s, t\}, \end{aligned}$$

28c $$\begin{aligned}&f_{uv} = \sum _{i \in \{1,\dots ,k\}} \pi _{uvi}  &   \forall (u,v) \in E, \end{aligned}$$

28d $$\begin{aligned}&\mathbf {\forall (u,v) \in E, \forall i \in \{1, \ldots , k\} :} \nonumber \\&\quad \pi _{uvi} \le f_{uv} x_{uvi} \end{aligned}$$

28e $$\begin{aligned}&\quad \pi _{uvi} \le w_i \end{aligned}$$

28f $$\begin{aligned}&\quad \pi _{uvi} \ge w_i - (1-x_{uvi})f_{uv} \end{aligned}$$

28g $$\begin{aligned}&\mathbf {\forall C \in {\mathcal {C}} \forall i \in \{1,\dots ,k\}:} \nonumber \\&\quad \sum _{(u,v) \in E(C)} x_{uvi} \ge |C| - M(1-\beta _{Ci}) \end{aligned}$$

28h $$\begin{aligned}&\quad \sum _{(u,v) \in E(C)} x_{uvi} - |C| + 1 - M\beta _{Ci} \le 0 \end{aligned}$$

28i $$\begin{aligned}&\quad \sum _{(u,v) \in \delta ^+(C) \setminus E(C)} x_{uvi} \ge \beta _{Ci}, \end{aligned}$$

28j $$\begin{aligned}&w_i \in {\mathbb {Z}}^+  &   \forall i \in \{1,\dots ,k\}, \end{aligned}$$

28k $$\begin{aligned}&x_{uvi} \in \{0,1\}  &   \forall (u,v) \in E, \forall i \in \{1,\dots ,k\}, \end{aligned}$$

28l $$\begin{aligned}&\pi _{uvi} \in {\mathbb {Z}}^+ \cup \{0\}  &   \forall (u,v) \in E, \forall i \in \{1,\dots ,k\}, \end{aligned}$$

28m $$\begin{aligned}&\beta _{Ci} \in \{0,1\}  &   \forall C \in {\mathcal {C}}, \forall i \in \{1,\dots ,k\}. \end{aligned}$$

## Full ILP formulation for *k*-Flow decomposition into walks (*k*-FDW): $x_{uvi}$ as binary variables

29a $$\begin{aligned}&\mathbf {\forall i \in \{1, \ldots , k\}:} \nonumber \\&\quad \sum _{(s,v) \in E} x_{svi} \le 1 \end{aligned}$$

29b $$\begin{aligned}&\quad \sum _{(u,v) \in E} x_{uvi} - \sum _{(v,w) \in E} x_{vwi} = 0  &   \forall v \in V \setminus \{s, t\}, \end{aligned}$$

29c $$\begin{aligned}&f_{uv} = \sum _{i \in \{1,\dots ,k\}} \pi _{uvi}  &   \forall (u,v) \in E, \end{aligned}$$

29d $$\begin{aligned}&\mathbf {\forall (u,v) \in E, \forall i \in \{1,\dots ,k\}:} \nonumber \\&\quad \pi _{uvi} \le f_{uv} x_{uvi} \end{aligned}$$

29e $$\begin{aligned}&\quad \pi _{uvi} \le w_i \end{aligned}$$

29f $$\begin{aligned}&\quad \pi _{uvi} \ge w_i - (1-x_{uvi})f_{uv} \end{aligned}$$

29g $$\begin{aligned}&\mathbf {\forall v \in V \setminus \{s\}, \forall i \in \{1,\dots ,k\}:} \nonumber \\&\quad \sum _{(u,v) \in E} x_{uvi} \ge d_{vi} \end{aligned}$$

29h $$\begin{aligned}&\quad \sum _{(u,v) \in E} x_{uvi} \le \sum _{(u,v) \in E} \phi _{uvi} \end{aligned}$$

29i $$\begin{aligned}&\quad \sum _{(u,v) \in E} x_{uvi} \le |E| \sum _{(u,v) \in E} y_{uvi} \end{aligned}$$

29j $$\begin{aligned}&\quad \sum _{(u,v) \in E} y_{uvi} \le 1 \end{aligned}$$

29k $$\begin{aligned}&\mathbf {\forall i \in \{1, \ldots , k\}:} \nonumber \\&\quad x_{uvi} \ge y_{uvi}  &   \forall (u,v) \in E, \end{aligned}$$

29l $$\begin{aligned}&\quad d_{si} = 1 \end{aligned}$$

29m $$\begin{aligned}&\mathbf {\forall (u,v) \in E, \forall i \in \{1,\dots ,k\}:} \nonumber \\&\quad \phi _{uvi} \le n y_{uvi} \end{aligned}$$

29n $$\begin{aligned}&\quad \phi _{uvi} \ge -n y_{uvi} \end{aligned}$$

29o $$\begin{aligned}&\quad \phi _{uvi} \le (d_{vi} - d_{ui}) + (1-y_{uvi})n \end{aligned}$$

29p $$\begin{aligned}&\quad \phi _{uvi} \ge (d_{vi} - d_{ui}) - (1-y_{uvi})n \end{aligned}$$

29q $$\begin{aligned}&w_i \in {\mathbb {Z}}^+  &   \forall i \in \{1,\dots ,k\}, \end{aligned}$$

29r $$\begin{aligned}&x_{uvi} \in \{0,1\}  &   \forall (u,v) \in E, \forall i \in \{1,\dots ,k\}, \end{aligned}$$

29s $$\begin{aligned}&\pi _{uvi} \in {\mathbb {Z}}^+ \cup \{0\}  &   \forall (u,v) \in E, \forall i \in \{1,\dots ,k \end{aligned}$$

29t $$\begin{aligned}&d_{vi} \in {\mathbb {Z}}^+  &   \forall v \in V, \forall i \in \{1,\dots ,k\}, \end{aligned}$$

29u $$\begin{aligned}&y_{uvi} \in \{0,1\}  &   \forall (u,v) \in E, \forall i \in \{1,\dots ,k\}, \end{aligned}$$

29v $$\begin{aligned}&\phi _{uvi} \in {\mathbb {Z}}  &   \forall (u,v) \in E, \forall i \in \{1,\dots ,k\}. \end{aligned}$$

## Full ILP formulation for *k*-Flow decomposition into walks (*k*-FDW): $x_{uvi}$ as an integer variables

30a $$\begin{aligned}&\mathbf {\forall i \in \{1, \ldots , k\}:} \nonumber \\&\quad \sum _{(s,v) \in E} x_{svi} \le 1 \end{aligned}$$

30b $$\begin{aligned}&\quad \sum _{(u,v) \in E} x_{uvi} - \sum _{(v,w) \in E} x_{vwi} = 0  &   \forall v \in V \setminus \{s, t\}, \end{aligned}$$

30c $$\begin{aligned}&\quad w_i = \sum _{j \in \{0,\dots ,\lfloor \log _2({\overline{w}})\rfloor \}} 2^j \zeta _{ij} \end{aligned}$$

30d $$\begin{aligned}&f_{uv} = \sum _{i \in \{1,\dots ,k\}} \sum _{j \in \{0,\dots ,\lfloor \log _2({\overline{w}})\rfloor \}} 2^j\varphi _{uvij}  &   \forall (u,v) \in E, \end{aligned}$$

30e $$\begin{aligned}&\mathbf {\forall (u,v) \in E, \forall i \in \{1,\dots ,k\}, \forall j \in \{0,\dots ,\lfloor \log _2({\overline{w}})\rfloor \} :} \nonumber \\&\quad \varphi _{uvij} \le f_{uv} \zeta _{ij}  &   \end{aligned}$$

30f $$\begin{aligned}&\quad \varphi _{uvij} \le x_{uvi} \end{aligned}$$

30g $$\begin{aligned}&\quad \varphi _{uvij} \ge x_{uvi} - (1-\zeta _{ij})f_{uv}&\end{aligned}$$

30h $$\begin{aligned}&\mathbf {\forall v \in V \setminus \{s\}, \forall i \in \{1,\dots ,k\}:} \end{aligned}$$

30i $$\begin{aligned}&\quad \sum _{(u,v) \in E} x_{uvi} \ge d_{vi} \end{aligned}$$

30j $$\begin{aligned}&\quad \sum _{(u,v) \in E} x_{uvi} \le \sum _{(u,v) \in E} \phi _{uvi} \end{aligned}$$

30k $$\begin{aligned}&\quad \sum _{(u,v) \in E} x_{uvi} \le |E| \sum _{(u,v) \in E} y_{uvi} \end{aligned}$$

30l $$\begin{aligned}&\quad \sum _{(u,v) \in E} y_{uvi} \le 1 \end{aligned}$$

30m $$\begin{aligned}&\mathbf {\forall (u,v) \in E, \forall i \in \{1,\dots ,k\}:} \end{aligned}$$

30n $$\begin{aligned}&x_{uvi} \ge y_{uvi} \end{aligned}$$

30o $$\begin{aligned}&\quad \phi _{uvi} \le n y_{uvi} \end{aligned}$$

30p $$\begin{aligned}&\quad \phi _{uvi} \ge -n y_{uvi} \end{aligned}$$

30q $$\begin{aligned}&\quad \phi _{uvi} \le (d_{vi} - d_{ui}) + (1-y_{uvi})n \end{aligned}$$

30r $$\begin{aligned}&\quad \phi _{uvi} \ge (d_{vi} - d_{ui}) - (1-y_{uvi})n \end{aligned}$$

30s $$\begin{aligned}&\mathbf {\forall i \in \{1, \ldots , k\}:} \nonumber \\&\quad d_{si} = 1 \end{aligned}$$

30t $$\begin{aligned}&\quad w_i \in {\mathbb {Z}}^+ \end{aligned}$$

30u $$\begin{aligned}&\quad x_{uvi} \in {\mathbb {Z}}^+ \cup \{0\}  &   \forall (u,v) \in E,\end{aligned}$$

30v $$\begin{aligned}&\quad d_{vi} \in {\mathbb {Z}}^+ \cup \{0\}  &   \forall v \in V,\end{aligned}$$

30w $$\begin{aligned}&\quad y_{uvi} \in \{0,1\}  &   \forall (u,v) \in E, \end{aligned}$$

30x $$\begin{aligned}&\quad \phi _{uvi} \in {\mathbb {Z}}  &   \forall (u,v) \in E,\end{aligned}$$

30y $$\begin{aligned}&\mathbf {\forall i \in \{1,\dots ,k\}, \forall j \in \{0,\dots ,\lfloor \log _2({\overline{w}})\rfloor \},:} \nonumber \\&\quad \zeta _{ij} \in \{0,1\} \end{aligned}$$

30z $$\begin{aligned}&\quad \varphi _{uvij} \in {\mathbb {Z}}^+ \cup \{0\}  &   \forall (u,v) \in E. \nonumber \\&{\overline{w}} = \max _{(u,v) \in E} f_{uv} \end{aligned}$$
